# HLA-A*23 Is Associated With Lower Odds of Acute Retroviral Syndrome in Human Immunodeficiency Virus Type 1 Infection: A Multicenter Sub-Saharan African Study

**DOI:** 10.1093/ofid/ofae129

**Published:** 2024-03-13

**Authors:** Lovisa Lindquist, William Kilembe, Etienne Karita, Matt A Price, Anatoli Kamali, Pontiano Kaleebu, Jianming Tang, Susan Allen, Eric Hunter, Jill Gilmour, Sarah L Rowland-Jones, Eduard J Sanders, Amin S Hassan, Joakim Esbjörnsson

**Affiliations:** Lund University Centre, Lund University, Lund, Sweden; Department of Translational Medicine, Lund University, Lund, Sweden; Rwanda/Zambia HIV Research Group, Kigali, Rwanda and Lusaka, Zambia; Rwanda/Zambia HIV Research Group, Kigali, Rwanda and Lusaka, Zambia; International AIDS Vaccine Initiative, New York, New York, USA; Department of Epidemiology and Biostatistics, University of California, San Francisco, San Francisco, California, USA; International AIDS Vaccine Initiative, Nairobi, Kenya; Medical Research Council/Uganda Virus Centre Research Institute, Entebbe, Uganda; London School of Hygiene and Tropical Medicine, London, United Kingdom; Department of Medicine, University of Alabama at Birmingham, Birmingham, Alabama, USA; Rwanda/Zambia HIV Research Group, Kigali, Rwanda and Lusaka, Zambia; Emory Vaccine Center, Emory University, Atlanta, Georgia, USA; Rwanda/Zambia HIV Research Group, Kigali, Rwanda and Lusaka, Zambia; Emory Vaccine Center, Emory University, Atlanta, Georgia, USA; International AIDS Vaccine Initiative, New York, New York, USA; Human Immunology Laboratory, International AIDS Vaccine Initiative, London, United Kingdom; Nuffield Department of Clinical Medicine, University of Oxford, Oxford, United Kingdom; Nuffield Department of Clinical Medicine, University of Oxford, Oxford, United Kingdom; Aurum Institute, Johannesburg, South Africa; Lund University Centre, Lund University, Lund, Sweden; Department of Translational Medicine, Lund University, Lund, Sweden; Kenya Medical Research Institute/Wellcome Trust Research Programme, Kilifi, Kenya; Lund University Centre, Lund University, Lund, Sweden; Department of Translational Medicine, Lund University, Lund, Sweden; Nuffield Department of Clinical Medicine, University of Oxford, Oxford, United Kingdom

**Keywords:** acute HIV infection, acute retroviral syndrome, Africa, HLA, KIR

## Abstract

The role of human leukocyte antigen (HLA) class I and killer immunoglobulin-like receptor molecules in mediating acute retroviral syndrome (ARS) during human immunodeficiency virus type 1 (HIV-1) infection is unclear. Among 72 sub-Saharan African adults, HLA-A*23 was associated with lower odds of ARS (adjusted odds ratio, 0.10 [95% confidence interval, .01–.48]; *P* = .009), which warrants further studies to explore its role on HIV-1–specific immunopathogenesis.

Acute human immunodeficiency virus type 1 (HIV-1) infection (AHI) is characterized by high viral load and innate immune activation [1]. The innate immune response involves natural killer (NK) cells, which use killer immunoglobulin-like receptors (KIRs) to identify and eradicate virus-infected cells by interacting with human leukocyte antigen (HLA) class I molecules [2]. As a result of the virus–host interactions during AHI, 56%–78% of people with HIV-1 manifest signs and symptoms within the first weeks of infection, generally referred to as acute retroviral syndrome (ARS) [3–5]. High HIV-1 viral load, low CD4^+^ T-cell count, and a stronger innate immune response during AHI have been associated with ARS [3–5].

In the context of HIV-1 pathogenesis, HLA/KIR alleles have been extensively studied. HLA-B*57 has been associated with low viral load, high CD4^+^ T-cell count, and slower disease progression [6]. In contrast, KIR2DS2, HLA-B*35-Px, and homozygosity at HLA class I loci have been associated with rapid disease progression [6, 7]. In addition, HLA-A*23 and HLA-C*07 have been associated with lower viral replicative capacity (VRC) in HIV-1 subtype A1 infections [8]. However, the role of HLA/KIR alleles in mediating retroviral syndrome during AHI has not been established. In this study, we aimed to explore associations between a wide panel of HLA/KIR alleles and ARS among adults in a multicenter AHI cohort from sub-Saharan Africa.

## METHODS

### Study Population

A retrospective analysis of data from the International AIDS Vaccine Initiative (IAVI) protocol C cohort was performed [9]. Adults (≥18 years) diagnosed with AHI (defined as a positive HIV-1 RNA or p24 antigen test, but negative antibody test, indicating Fiebig stage I/II; and sample collection 10–21 days since the estimated date of infection [EDI]) between 2006 and 2011 in 1 of 4 sites in sub-Saharan Africa (Kilifi, Kenya; Masaka, Uganda; Kigali, Rwanda; or Lusaka, Zambia) were eligible. Individual-level data included age, sex, study site, EDI, risk group (heterosexual and men who have sex with men), HIV-1 subtype, and HIV-1 viral load (first observation within 21 days of EDI), HLA/KIR profiles, and AHI symptom data [9].

### Patient Consent Statement

All participants provided written informed consent. The IAVI protocol C sites received approvals from the ethics review boards of the respective countries: the Kenya Medical Research Institute Ethical Review Committee, the Kenyatta National Hospital Ethical Review Committee of the University of Nairobi, the Rwanda National Ethics Committee, the Uganda Virus Research Institute Science and Ethics Committee, the Uganda National Council of Science and Technology, and the University of Zambia Research Ethics Committee [10].

### HLA/KIR Typing

HLA class I and KIR alleles were genotyped from genomic DNA, as described previously [11]. HLA alleles were analyzed at 2-digit resolution (allele group). For KIR genotyping, the presence or absence of individual genes was determined by polymerase chain reaction. Allele positivity was defined as presence on 1 (heterozygous) or both (homozygous) chromosomes.

### Acute Retroviral Syndrome

Eleven signs and symptoms including fever, fatigue, headache, night sweats, myalgia, pharyngitis, lymphadenopathy, anorexia, diarrhea, oral ulcers, and skin rash were screened and documented using standardized questionnaires within 2–6 weeks of the EDI [10]. Latent class analysis (LCA, a probabilistic modeling approach for clustering of categorical data) was used to group participants based on the number of symptoms and unobserved linkages between them, as previously demonstrated [5]. The best-fit model for 2–6 classes was explored and determined based on the model with the lowest Bayesian information criterion (BIC). LCA was performed in RStudio (package: poLCA).

### Data Analysis

The distribution of HLA/KIR alleles and AHI symptoms were presented as proportions of the study population. The performance of LCA to disentangle the study population by ARS based on the symptom data was validated using 95% confidence intervals (CIs) and Fisher's exact tests. Proportions of participants with and without specific HLA/KIR alleles by ARS were compared using Fisher's exact tests, and correction for multiple comparisons was performed using the Benjamini-Hochberg method. Alleles present or absent at <5% of the study population were excluded from further analysis due to sample size limitations. Individual HLA/KIR alleles were analyzed in a bivariate logistic regression model, and alleles with *P* < .05 for associations with ARS were carried forward to a multivariate logistic regression model to control for age, sex, study site, risk group, HIV-1 subtype, and HIV-1 viral load. HLA/KIR alleles with *P* < .05 in the multivariate logistic regression model were considered significantly associated with ARS. All data analyses were performed in RStudio (packages: tidyverse, stats, DescTools), and graphs were reproduced using GraphPad Prism (version 9.3.1).

## RESULTS

### Study Population

Of 74 eligible participants, 2 were excluded for missing HLA/KIR (n = 1) or ARS (n = 1) data. The mean age of the remaining 72 participants was 29 (standard deviation, 8) years. A majority was male (n = 58 [80.6%]), heterosexual (n = 45 [62.5%]), and infected with HIV-1 subtype A1 (n = 45 [62.5%]) (Table 1).

**Table 1.** Characteristics of Eligible Participants Diagnosed With Acute Human Immunodeficiency Virus Type 1 Infection From Kenya, Rwanda, Uganda, and Zambia (N = 72)

Characteristic	Frequency (%)
Age, y, mean (SD)	29 (8)
Age group, y	
18–24	23 (31.9)
25–34	35 (48.6)
35–44	8 (11.1)
45–54	6 (8.3)
Sex	
Female	14 (19.4)
Male	58 (80.6)
Risk group	
Heterosexual	45 (62.5)
MSM	27 (37.5)
Country of enrollment	
Kenya	31 (43.1)
Rwanda	13 (18.1)
Uganda	13 (18.1)
Zambia	15 (20.8)
HIV-1 subtype	
A1	45 (62.5)
A2D	1 (1.4)
C	18 (25.0)
D	7 (9.7)
G	1 (1.4)
HIV-1 viral load^a^ group, log_10_ cpm	
>6.0	20 (27.8)
<6.0	30 (41.7)
Missing	22 (30.6)
HIV-1 viral load^a^, log_10_ cpm, mean (SD)	5.95 (1.15)

Abbreviations: cpm, RNA copies per milliliter of plasma; HIV-1, human immunodeficiency virus type 1; MSM, men who have sex with men; SD, standard deviation.

^a^HIV-1 viral load based on the first documented observation after the estimated date of infection.

### HLA/KIR Profiles

**Figure 1.**
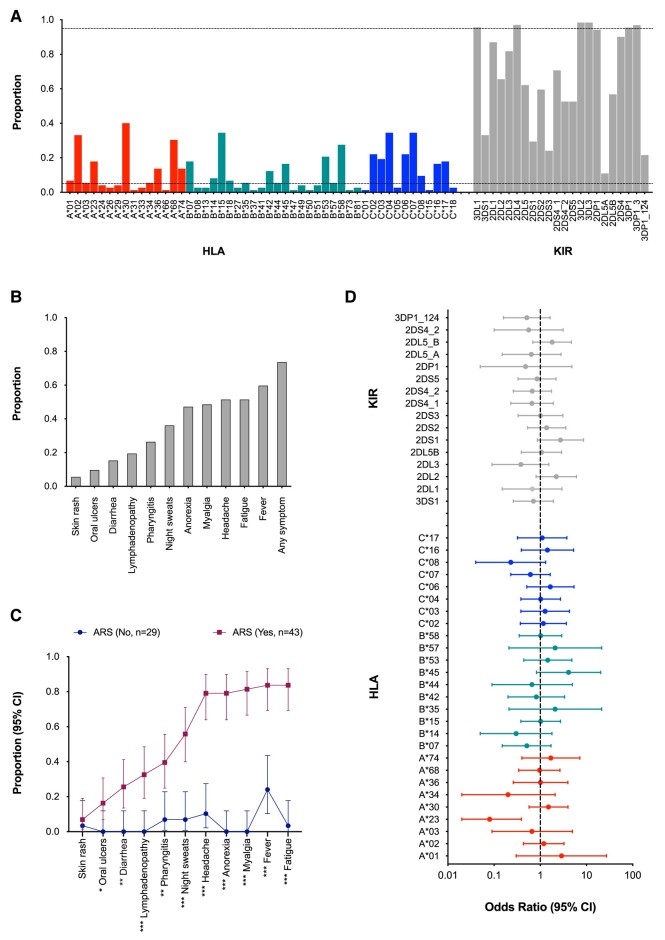
Distributions of human leukocyte antigen (HLA)/killer immunoglobulin-like receptor (KIR) alleles and acute human immunodeficiency virus type 1 infection (AHI) symptoms in the study population and associations with acute retroviral syndrome (ARS) (N = 72). *A*, Distribution of HLA/KIR alleles in the study population. HLA-A (n = 15), HLA-B (n = 22), HLA-C (n = 12), and KIR (n = 22) variants were explored. Alleles with a frequency of >5% or <95% (dotted lines) were subjected to association analyses. *B*, Distribution of AHI symptoms, showing the proportion of each symptom before stratification by ARS. *C*, Distribution of AHI symptoms by ARS, defined using latent class analysis (LCA). LCA was used to stratify the participants into groups based on the experienced symptoms and unobserved linkages between them. Participants were clustered into those with ARS (n = 43) and those without ARS (n = 29). Fisher's exact tests was used to determine significant differences: *P < .05, **P < .01, ***P < .001. *D*, Associations between HLA/KIR alleles and ARS, as illustrated in a forest plot summarizing associations between HLA/KIR alleles included in the analysis with ARS, presented using odds ratios and 95% confidence intervals (CIs).

HLA-A (n = 15), HLA-B (n = 22), HLA-C (n = 12), and KIR (n = 22) variants were profiled. The most prevalent HLA-A, -B, and -C alleles were HLA-A*30 (40.3%), HLA-B*15 (34.7%), and HLA-C*04 and HLA-C*07 (both at 34.7%). The most prevalent KIR genes were *KIR3DL2* and *KIR3DL3* (both at 98.6%) (Figure 1*A*).

### AHI Symptoms and ARS

The median number of symptoms was 4 (interquartile range [IQR], 0–6). Fifty-three (73.6%) participants reported at least 1 symptom. The most and least reported symptoms were fever (59.7%) and skin rash (5.6%) (Figure 1*B*). A 2-class LCA model performed best (BIC = 777.6) compared to a 3-class (BIC = 786.6), 4-class (BIC = 821.7), 5-class (BIC = 859.2), or 6-class (BIC = 899.2) model, and differentiated the participants into those with ARS (n = 43 [59.7%]; median number of symptoms: 5 [IQR, 4–8]) and without ARS (n = 29 [40.3%]; median number of symptoms, 0 [IQR, 0–1]). In a validation analysis, all symptoms except skin rash (*P* = .644, Fisher exact test) were significantly higher in the ARS group (Figure 1*C*).

### Associations Between HLA/KIR Alleles and ARS

The prevalence of ARS was lower among participants with HLA-A*23 compared to those without (15.4% vs 69.5%, *P* = .033, Fisher's exact tests with Benjamini-Hochberg correction; Supplementary Figure 1). Furthermore, participants with HLA-A*23 had lower odds of ARS compared to those without (Figure 1*D*), which was confirmed even after controlling for age, sex, risk group, HIV-1 subtype, and HIV-1 viral load (adjusted odds ratio, 0.10 [95% CI, .01–.48]; *P* = .009; Supplementary Table 1). There was no association between any of the other HLA molecules or KIR genes and ARS (Figure 1*D*).

## DISCUSSION

HLA-A*23 was associated with lower odds of ARS, independent of sex, age, risk group, HIV-1 subtype, and HIV-1 viral load. ARS is mediated by a cytokine storm, and specifically, high levels of interferon gamma (IFN-γ)–induced protein-10 (IP-10) have been observed during AHI [1, 5]. IP-10 is secreted by innate immune cells in response to IFN-γ, which is released by activated NK cells [12, 13], and activation of NK cells is influenced by the interaction with HLA class I molecules [2]. In addition, HLA-A*23, like other HLA alleles, possesses the HLA-Bw4-80I motif, a ligand for the inhibitory receptor KIR3DL1 expressed on NK cells [14]. It is therefore possible that if HLA-A*23 is sufficiently expressed at the cell surface to prevent NK cell activation, for example due to impaired downregulation by HIV-1 [15], the cytokine storm and subsequent manifestation of ARS may be diminished. Studies investigating associations between HLA-A*23 and specific cytokines and chemokines, as well as between HLA-A*23 and NK cell activation, are therefore warranted.

Our finding adds to the literature on associations between HLA-A*23, ARS, HIV-1 subtypes, VRC, and disease progression. Existing literature provides conflicting results on whether ARS associates with faster or slower disease progression. Whereas 2 studies associated ARS with rapid disease progression [3, 4], other studies demonstrated that individuals infected with subtype A1 had increased ARS [10] and slower disease progression [16], indicating that ARS may be associated with slower disease progression. Since HLA-A*23 is associated with lower VRC [8], which is associated with slower disease progression [17], it is reasonable to hypothesize that HLA-A*23 is associated with slower disease progression, potentially mediated by both lower VRC and ARS. However, elaborate studies are warranted to clearly elucidate associations between HLA-A*23, a common allele in some sub-Saharan African settings [18], with disease progression, while controlling for confounding factors. Further exploration of the influence and mechanisms of action of HLA-A*23 on the early immune responses and subsequent long-term effects could enable the identification of peptides for priming immune responses in attempts to reduce the inflammatory burden and mitigate disease progression.

Our study is not without limitations. Given the small sample size, it is possible that associations between other HLA/KIR alleles and ARS could have been identified in a larger study population. However, there were no borderline associations observed for any one of the other HLA/KIR alleles to suggest that an increase in the sample size may have yielded any different results. Because of the small sample size, the analysis of HLA alleles was limited to 2-digit resolution, and potential effects of hetero- and homozygosity were not explored. Yet, the study population comprised male and female volunteers, multiple subtypes, and 2 key risk groups, thereby representing a good degree of the diversity observed in the general HIV-1–infected populations.

In conclusion, HLA-A*23 was associated with lower odds of ARS among adults from sub-Saharan Africa. This observation increases our understanding of the role of HLA/KIR alleles in mediating retroviral syndrome during AHI. Further studies to disentangle the complex virus-host interactions during the establishment of HIV-1 infection and the role of HLA-A*23 on HIV-1–specific immune activation and disease pathogenesis are warranted to contribute to the evidence base needed for the design of effective prophylactic or therapeutic interventions.

## Supplementary Material

ofae129_Supplementary_Data

## Supplementary Data

Supplementary materials are available at *Open Forum Infectious Diseases* online. Consisting of data provided by the authors to benefit the reader, the posted materials are not copyedited and are the sole responsibility of the authors, so questions or comments should be addressed to the corresponding author.

## Notes

***Acknowledgments.*** We are grateful to IAVI for supporting HIV-1 research studies and capacity-building initiatives in Kenya, Uganda, Rwanda, and Zambia. We are also grateful to staff and participants from IAVI's protocol C sites in Africa, without whom this work would not have been possible.

***Author contributions.*** J. E. and A. S. H. conceptualized the study and designed the analysis plan. L. L. carried out the analysis and drafted the manuscript. W. K., E. K., M. A. P., A. K., P. K., J. T., S. A., E. H., and J. G. generated the data. W. K., E. K., M. A. P., A. K., P. K., S. A., E. H., J. G., S. R.-J., E. J. S., A. S. H., and J. E. reviewed the manuscript and provided feedback. All authors approved the final draft of the manuscript for submission.

***Financial support*.** This project was made possible in part by the generous support of the American people through the United States Agency for International Development. This work was also supported in part by funding from the Swedish Research Council (grant number 2016-01417 and 2020-06262), and the Swedish Society for Medical Research (grant number SA-2016). A. S. H. was supported by a training fellowship from the Wellcome Trust (award number 209294/Z/17/Z).
