# Supplementary material for: HLA-A*23 Is Associated With Lower Odds of Acute Retroviral Syndrome in Human Immunodeficiency Virus Type 1 Infection: A Multicenter Sub-Saharan African Study
Source: Open Forum Infect Dis. 2024 Mar 13;11(4):ofae129. doi: 10.1093/ofid/ofae129 (PMC10977907; doi:10.1093/ofid/ofae129)
Supplement: ofae129_Supplementary_Data [file ofae129_supplementary_data.docx]

**HLA-A*23 IS ASSOCIATED WITH LOWER ODDS OF ACUTE RETROVIRAL SYNDROME IN HIV-1 INFECTION: A MULTICENTRE SUB-SAHARAN AFRICAN STUDY**

Lovisa Lindquist (1), William Kilembe (2), Etienne Karita (2), Matt A. Price (3, 4), Anatoli Kamali (3), Pontiano Kaleebu (5), Jianming Tang (6), Susan Allen (2, 7), Eric Hunter (2, 7), Jill Gilmour (3, 8), Sarah L. Rowland-Jones (9), Eduard J. Sanders (9, 10), Amin S. Hassan (1, 11) *, and Joakim Esbjörnsson (1, 9)§*

*Authors with equal contribution

Author affiliations: (1) Department of Translational Medicine, Lund University, Sweden; (2) Rwanda/Zambia HIV Research Group, Kigali, Rwanda and Lusaka Zambia; (3) IAVI New York, USA and Nairobi, Kenya; (4) UCSF Department of Epidemiology and Biostatistics, San Francisco, CA, USA; (5) Medical Research Council/Uganda Virus Centre Research Institute, Uganda and London School of Hygiene and Tropical Medicine, London UK; (6) Department of Medicine, University of Alabama at Birmingham, Birmingham, AL, USA; (7) Emory Vaccine Center, Emory University, Atlanta, GA, USA; (8) IAVI Human Immunology Laboratory, London, UK; (9) Nuffield Department of Clinical Medicine, University of Oxford, UK; (10) Aurum Institute, Johannesburg, South Africa, and (11) KEMRI/Wellcome Trust Research Programme, Kilifi, Kenya.

**§Corresponding Author:**

Department of Translational Medicine, Lund University

221 84 Lund, Sweden

Email: joakim.esbjornsson@med.lu.se

**Supplementary Figure 1. Distribution of HLA/KIR alleles by ARS.** Participants were stratified into groups with ARS (N=43) and without ARS (N=29) using latent class analysis. The proportions and 95% CI of participants experiencing ARS in presence (filled) and absence (hollow) of each HLA/KIR allele are presented. Graphs and statistical analyses were performed in RStudio (packages: poLCA, tidyverse, and stats). *Abbreviations:* ARS=acute retroviral syndrome, CI=confidence intervals, HLA=human leukocyte antigen, KIR=killer immunoglobulin-like receptor.

**
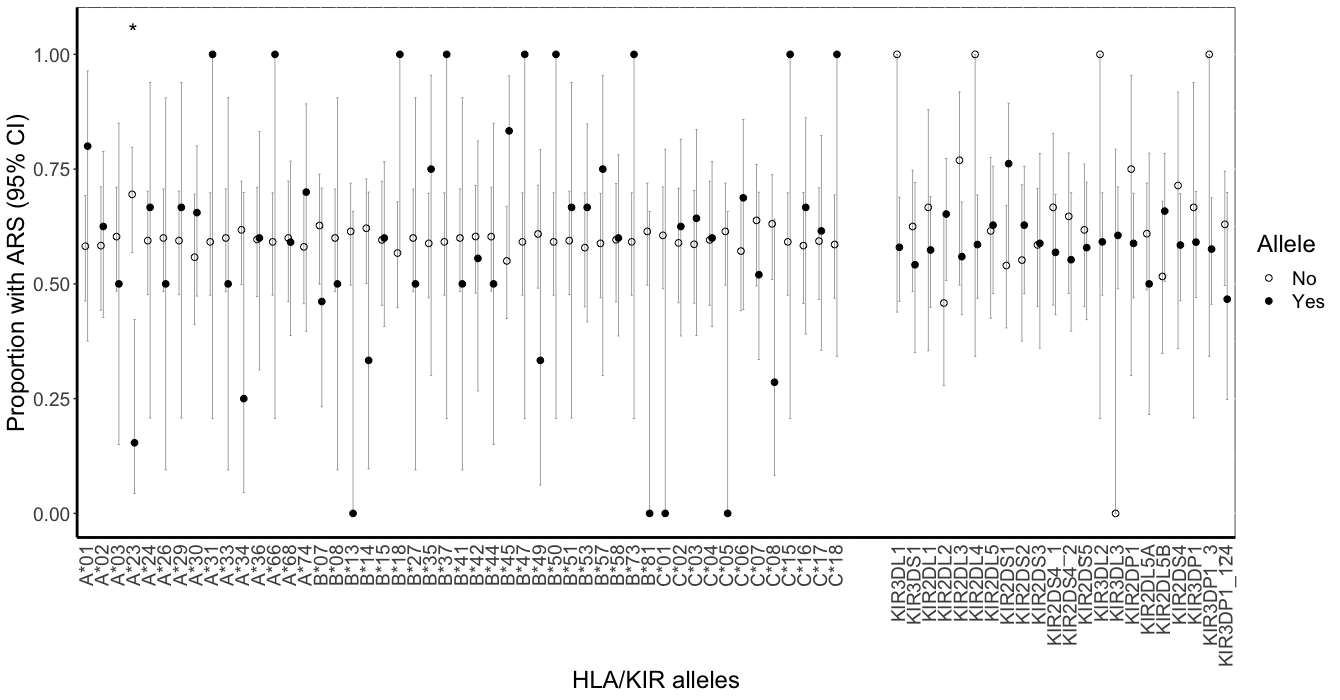
**

**Supplementary Table 1.** **Control of covariates with multivariate logistic regression models.** Table summarising bivariate and multivariate logistic regression analyses of data to describe associations between HLA/KIR alleles and ARS, controlling for covariates using data from Kenya, Uganda, Rwanda, and Zambia (N=72). The analyses were performed in RStudio (package: stats).

| Characteristics | | ARS, N (%) | Crude OR (95% CI) | P-value | Adj. OR (95% CI) | P-value |
| --- | --- | --- | --- | --- | --- | --- |
| HLA-A*23 | Negative | 41/59 (69.5) | Ref. |  | Ref. |  |
|  | Positive | 2/13 (15.4) | 0.08 (0.02-0.39) | 0.002 | 0.10 (0.02-0.57) | 0.009 |
| Risk group | HET | 21/45 (46.7) | Ref. |  | Ref. |  |
|  | MSM | 22/27 (81.5) | 5.03 (1.62-15.63) | 0.005 | 1.61 (0.36-7.22) | 0.532 |
| Subtype | Other | 10/27 (37.0) | Ref. |  | Ref. |  |
|  | A1 | 33/45 (73.3) | 4.68 (1.68-13.00) | 0.003 | 3.68 (1.12-12.05) | 0.031 |
| Sex | Female | 8/14 (57.1) | Ref. |  |  |  |
|  | Male | 35/58 (60.3) | 1.14 (0.34-3.72) | 0.827 |  |  |
| Age | - | - | 0.93 (0.87-0.99) | 0.030 | 0.96 (0.88-1.04) | 0.349 |
| HIV-1 viral load (log_10_ cpm) | min/6.0 cpm | 14/20 (70.0) | Ref. |  |  |  |
|  | 6.0+ cpm | 20/30 (66.7) | 0.86 (0.25-2.91) | 0.846 |  |  |
|  | Missing | 9/22 (40.9) | 0.29 (0.08-1.07 | 0.063 |  |  |
| HIV-1 viral load (log_10_) | | - | 0.95 (0.56-1.61) | 0.841 |  |  |

Covariates independently associated with ARS (p<0.05) in bivariate regression models were included in the multivariate regression model. Other subtypes include HIV-1 subtypes A2D (N=1), C (N=18), D (N=7), and G (N=1). *Abbreviations:* ARS=acute retroviral syndrome, OR=odds ratio, CI=confidence intervals, HLA=human leukocyte antigen, MSM=men who have sex with men, HET=heterosexual, cpm=RNA copies per millilitre of plasma.
